# Data of serial synchrotron crystallography of xylanase GH11 from *Thermoanaerobacterium saccharolyticum*

**DOI:** 10.1016/j.dib.2024.110055

**Published:** 2024-01-12

**Authors:** Ki Hyun Nam

**Affiliations:** College of General Education, Kookmin University, Seoul 02707, South Korea

**Keywords:** Serial synchrotron crystallography, Xylanase, Glycosyl hydrolase 11, Room temperature

## Abstract

The endo-1,4-β-xylanase GH11 from the hemicellulose-degrading bacterium *Thermoanaerobacterium saccharolyticum* (TsaGH11) has been characterized as a thermophilic enzyme. TsaGH11 exhibits its maximum activity at pH 5.0 and 70 °C, along with superior properties towards beechwood xylan, with a K_m_ of 12.9 mg mL⁻¹ and a K_cat_ of 34,015.3 s⁻¹. The room-temperature and cryogenic crystal structures of TsaGH11 were determined using serial synchrotron crystallography (SSX) and conventional macromolecular crystallography techniques, respectively. The high-resolution crystal structure of TsaGH11 was successfully determined, and the flexibility of the thumb domain at room temperature was elucidated. During SSX data collection, a high density of crystal samples in the sample holder led to an unprecedentedly high multi-crystal hit rate of ∼200 %. Data containing these multi-crystal hits will potentially be a valuable resource for developing indexing algorithms for multi-crystal hit patterns in serial crystallography (SX) data processing. To contribute to developing SX data processing, this paper provides detailed and specific information about the data collection and processing of TsaGH11 obtained through SSX experiments.

Specifications Table

**Table:** 

Subject	Biological sciences
Specific subject area	Structural Biology
Data format	Raw, Analyzed
Type of data	X-ray diffraction data, Table, Image, Graph, Figure
Data collection	Synchrotron: Pohang Light Source II (PLS-II) Beamline: 11C X-ray energy: 15.48 keV Photon flux: ∼2 × 10^11^ photons/second Beam size: 3.5 × 8 µm (full width at half maximum, vertical x horizontal) Sample delivery: fixed-taget sanning Sample holder: nylon mesh and enclosing film (NAM)-based sample holder Raster scan interval: 50 µm Detector: Pilatus3S 6 M (DECTRIS). Data acquisition: 10 Hz Data collection temperature: 26.5 ± 0.5 °
Data source location	Institution: Kookmin University City/Town/Region: Seoul Country: Republic of Korea
Data accessibility	1. Raw data diffraction images Repository name: ZENODO Data 1: Room-temperature structure of TsaGH11 - Digital Object Identifier: https://doi.org/10.5281/zenodo.7101471 - Direct URL to data: https://zenodo.org/records/7101471 Data 2: Room-temperature structure of TsaGH11-II - Digital Object Identifier: https://doi.org/10.5281/zenodo.7106300 - Direct URL to data: https://zenodo.org/records/7106300 2. Structure factor and coordinate Repository name: Protein Data Bank Data name: Room temperature structure of GH11 from Thermoanaerobacterium saccharolyticum by serial crystallography - PDB code: 8IH1 - Data identification number: https://doi.org/10.2210/pdb8IH1/pdb - Direct URL to data: https://www.rcsb.org/structure/8IH1
Related research article	I.J. Kim, S.R. Kim, K.H. Kim, U.T. Broushoer, K.H. Nam, Characterization and structural analysis of the endo-1,4-β-xylanase GH11 from the hemicellulose-degrading *Thermoanaerobacterium saccharolyticum* useful for lignocellulose saccharification, Scientific Reports, (2023) [1]https://doi.org/10.1038/s41598-023-44495-8

## 1 Value of the Data

• The diffraction data of TsaGH11 were collected using serial synchrotron crystallography.
• Multi-crystal hits on the diffraction data of TsaGH11 were observed and processed.
• Room-temperature structures of TsaGH11 were determined at a resolution of 1.75 Å.
• The SSX data containing the multi-crystal hits can be utilized as a valuable resource for developing data processing programs.

## 2 Background

Endo-1,4-β-xylanase GH11s are widely used in various industries, including feed, textile, chemical, pharmacy, and biorefinery [1,2]. The ongoing and crucial exploration for discovering new xylanases with high activity and stability from novel microorganism sources is essential for industrial applications [3,4]. Functional and structural analyses of xylanase GH11 from the hemicellulose-degrading bacterium *Thermoanaerobacterium saccharolyticum* (TsaGH11) have been conducted. This enzyme demonstrated high activity towards beechwood xylans with a k_cat_/K_m_ value of 2658.7 mL mg^−1^ s ^−^ ^1^ [1]. Crystal structures of TsaGH11 exhibited open and closed conformations of the substrate-binding cleft [1]. To better understand the molecular flexibility of TsaGH11, the room-temperature structure of TsaGH11 was determined by a serial synchrotron crystallography (SSX) experiment. The thumb domain of TsaGH11 displayed a disordered electron density map, indicating the high flexibility of the thumb domain at room temperature [1]. The diffraction images obtained from the SSX experiment exhibited a multi-crystal hit pattern, providing a potential resource for developing an indexing algorithm for multi-crystal hit patterns.

## 3 Data Description

**Fig. 1:**
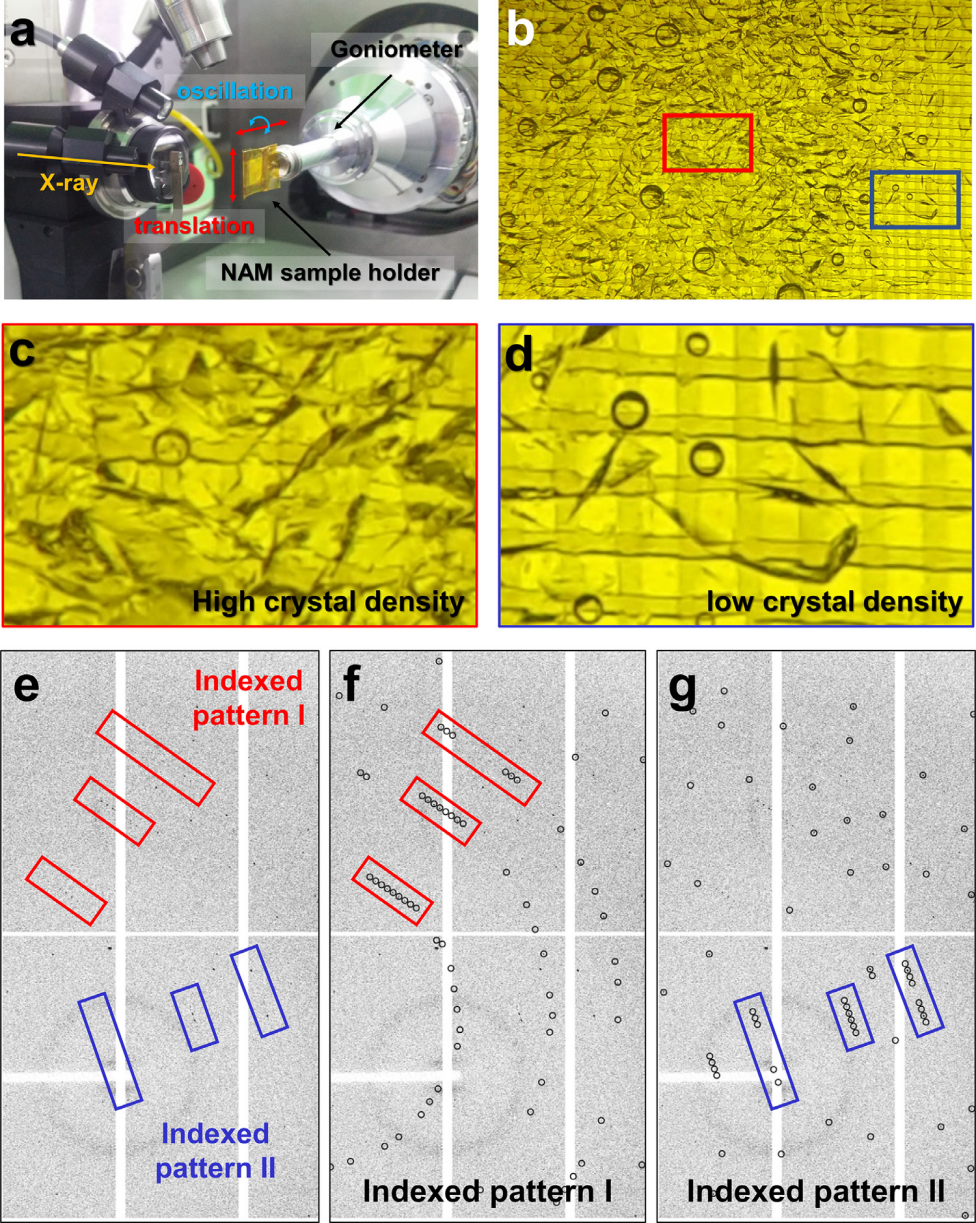
FT-SSX data collection of TsaGH11. (a) Experimental setup for the FT-SSX experiment. (b) Photograph of TsaGH11 crystals on NAM-based sample holder. Close-up view of the TsaGH11 crystals with (c) high and (d) low density on NAM-based sample holder. (e) Close-up view of multi-crystal indexing pattern from a single image of TsaGH11. (f,g) Two different indexed multicrystal lattices obtained from image in (e) The circles indicate the positions of Bragg peaks predicted by CrystFEL.

The research background, experimental procedure, characterization, and structural information of TsaGH11 have been reported [1]. To understand the function of TsaGH11, its crystal structure was determined using initial cryo-crystallography techniques. TsaGH11 features a typical β-jelly roll fold, resembling the right hand. The β-strand forms the finger and palm domains, constituting the active site cleft [1]. In the asymmetric unit, two TsaGH11 molecules exhibit open and closed conformations. The flexibility of the thumb domain in these two molecules appears to differ based on the influence of crystal packing. To observe the molecular flexibility characteristics of TsaGH11 at room temperature while minimizing the effects of radiation damage, SSX was performed [1] (Fig. 1a). Ultimately, it was confirmed that the thumb domain of TsaGH11 exhibits high molecular flexibility, as evidenced by the disordered electron map of the thumb domain at room temperature [1].

During the SSX data collection of TsaGH11, a high percentage of multi-hit crystal patterns were observed in the images collected. Due to the nature of serial crystallography (SX) experiments, multi-crystal hit rates are inevitable, and SX indexing programs have been developed to extract diffraction patterns from multiple crystals within a single image [5]. Consequently, successful multi-crystal indexing and high-resolution structure determination were achieved during TsaGH11 data processing. Meanwhile, the multi-crystal hit pattern of TsaGH11 is unprecedentedly high (∼200 %), potentially serving as a valuable resource for developing effective multi-crystal hit indexing algorithms in SX indexing programs. For the potential use of diffraction data from TsaGH11, detailed information on data collection and data processing are here reported.

In typical SX, the crystal is exposed to X-rays only once to minimize radiation damage [6]. Consequently, a large number of crystals are required to obtain three-dimensional crystal structure information [6]. The crystallization solution, which was crystallized using the hanging drop vapor diffusion method, was harvested with a pipette. The harvested crystal suspension (50 µl) was deposited into a nylon-mesh sample holder with an area of 8 × 8 mm (Fig. 1b–d). A sample holder containing high-density TsaGH11 crystals was mounted on a goniometer (Fig. 1a). To prevent overlapping X-ray exposure to the crystal sample, raster scans were performed in vertical and horizontal directions at 50 µm intervals based on the full width of the X-ray beam size. Finally, approximately 38,000 diffraction images were collected by performing the fixed-target scanning method. In the diffraction image, Bragg peaks were observed to diffract to over 1.6 Å. Most diffraction images contained two or more diffraction patterns (Fig. 1e). This multi-crystal hit rate is because that the crystals were deposited on the sample holder at high crystal density (Fig. 1c).

**Fig. 2:**
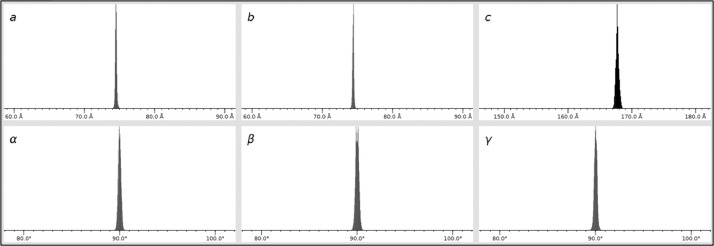
Unit cell distribution of TsaGH11 crystals from 66,580 indexed diffraction patterns.

Images without Bragg peaks or with low intensity are filtered using the Cheetah program, thereby reducing the subsequent data processing time and enabling efficient use of data storage space [7]. Total 32,250 images were obtained by filtering out images with no diffraction or weak diffraction intensities, resulting in a hit rate of approximately 85.11 %. During hit image indexing, the detector geometry was refined more than five times using *geoptimiser* in CrystFEL (Table 1). The hit image was processed using the CrystFEL program, and the unit cell information of TsaGH11 was entered and indexed with Xgandalf. Consequently, 32,302 images were indexed, achieving an indexing rate of 99.85 %. When the multi-crystal hit pattern was indexed, 65,980 diffraction patterns were obtained. As a result of image analysis, approximately two indexed diffraction patterns with the same crystal information were obtained from one image (Fig. 1e–g). This indicates an average of approximately two crystal diffraction patterns per image, with most diffraction images being multi-crystal diffraction patterns. Meanwhile, the hit and indexing rates introduced during data processing here vary depending on the data processing program or input parameter [8]. Accordingly, evaluating data quality based on final processed data processing statistics rather than the number of hit or indexing images is more appropriate. Indexing result showed that TsaGH11 crystals belonged to the tetragonal space group with an average unit cell of *a* = *b* = 73.11 Å, *c* = 165.42 Å, and α = β = γ = 90° from the distribution of unit cell parameter from 65,980 diffraction patterns (Fig. 2).

**Table 1:** Detector geometry information.

Detector geometry parameters	
photon_energy	12,659
clen	0.50
0/min_fs	0
0/max_fs	2462
0/min_ss	0
0/max_ss	2526
0/corner_x	−1232.89
0/corner_y	−1265.45
0/fs	+1.000000x − 0.000012y
0/ss	+0.000012x + 1.000000y
rigid_group_q0	0
rigid_group_a0	0
rigid_group_collection_quadrants	q0
rigid_group_collection_asics	a0
0/coffset	−0.000014

*This parameter was used for data processing, and the unfixed parameter value may change when performing additional detector geometry optimization.

**Fig. 3:**
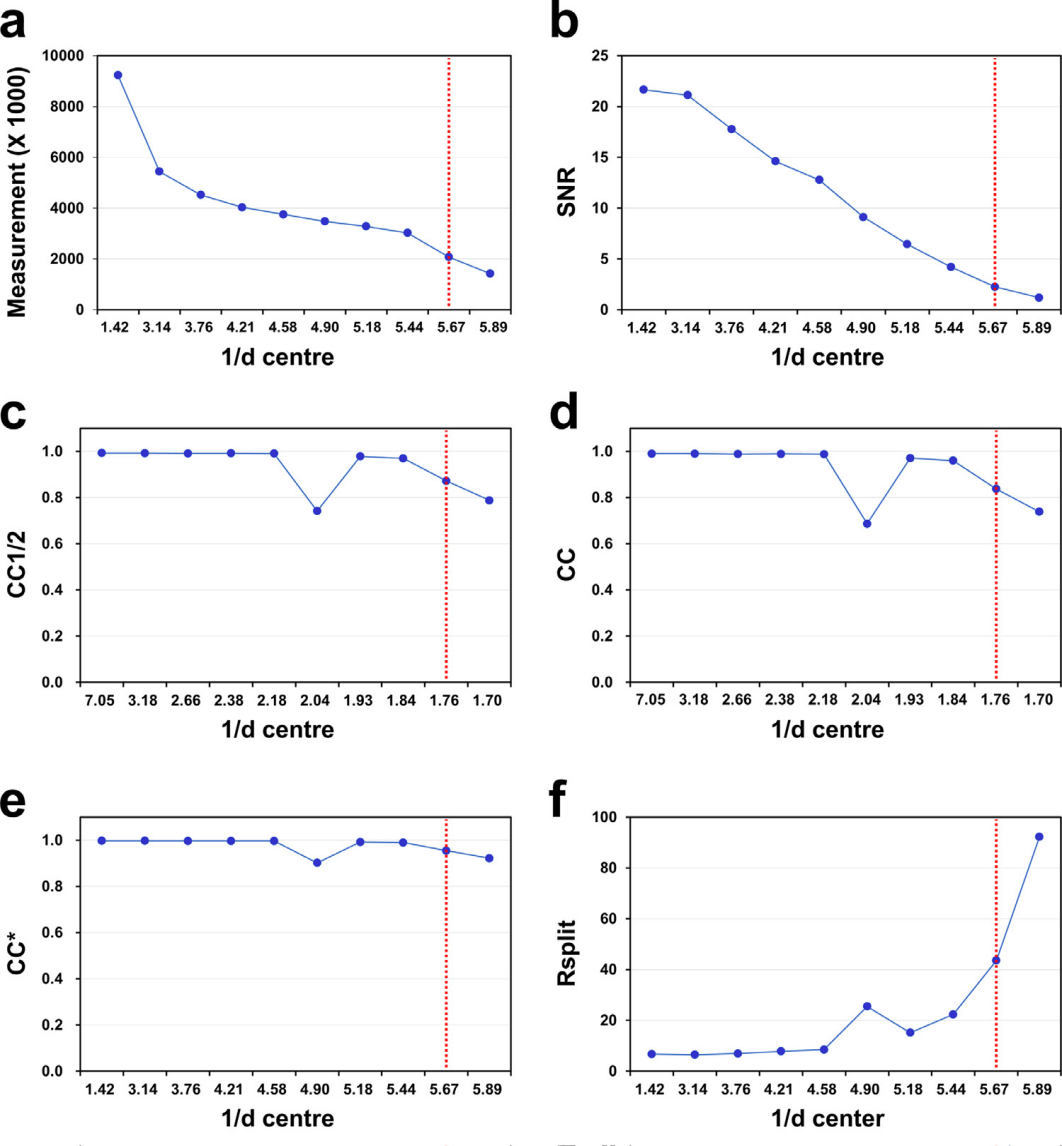
Data processing profiles of TsaGH11 collected by SSX experiment. Profiles of (a) measurement, (b) signal-to-noise ratio (SNR), (c) CC1/2, (d) CC, (e) CC*, and (f) Rsplit according to the 1/d center (nm). The resolution cut-off (1.75 Å) of the data in a previous report is indicated by a red-dotted line.

In a previous study, SSX data were processed to 1.75 Å, revealing measurement, completeness, redundancy, signal-to-noise ratio (SNR), CC*, and Rsplit values of 100, 775.8, 0.9965, 12.73, and 7.16, respectively (Table 2). The R_work_ and R_free_ of the final structure of SSX-TsaGH11 were 23.31 and 29.29, respectively. In previous experiment, the resolution cut-off of the crystal structure was considered based on the R-value. The high R-value of TsaGH11 compared with the resolution indicates increased flexibility. At the highest resolution shell, upon applying reasonable criteria of >1, >0.5, and <100 for SNR, CC1/2, and Rsplit, respectively, the resolution of SSX-TsaGH11 can be processed and used up to <1.7 Å. When applying the resolution cut-off in the range of 169–1.67 Å (highest resolution shell: 1.73–1.67 Å), the measurement, completeness, redundancy, SNR, CC, CC*, and Rsplit values were 56,633 (5553), 100.0 (100.0), 711.2 (256.7), 11.27 (1.19), 0.992 (0.738), 0.998 (0.921), and 7.27 (92.2), respectively (Fig. 3). This tentatively indicates that the crystal structure of TsaGH11 can be analyzed at a higher resolution than in previous studies. Merged TsaGH11 diffraction data show a general tendency for the data quality to deteriorate as the resolution increases. Meanwhile, at resolution of approximately 2 Å, CC, CC1/2, CC*, and Rspit values ​​appeared lower than would be expected from the observed tendency for the quality to decrease. In the collected images, no noticeable scattering that would degrade the diffraction quality was observed around 2 Å.

**Table 2:** Data collection and processing statistics of TsaGH11.

Data collection	SSX-TsaGH11	Processing up to 1.67 Å
Hit images	32,250	
Indexed images	32,203	
Diffraction pattern	65,986	
Space group	P4_3_2_1_2	
Cell dimensions (Å) a, b, c	74.47, 74.47, 167.74	
Resolution (Å)	172.4–1.75 (1.78–1.75)	169.49–1.67 (1.73–1.67)
No. of reflections	49,353 (4815)	56,633 (5553)
Completeness	100.0 (100.0)	100.0 (100.0)
Redundancy	775.8 (415.3)	711.2 (256.7)
I/σ(I)	12.73 (2.61)	11.27 (1.19)
CC	0.992 (0.857)	0.992 (0.738)
CC*	0.998 (0.960)	0.998 (0.921)
Rsplit^a^	7.16 (37.46)	7.27 (92.2)

The highest resolution shell is shown in parentheses.

a $\text{Rsplit}=\left(1\;/\;\sqrt{2}\right)\cdot \frac{\sum_{+hkl}\left|I_{hkl}^{even}-I_{hkl}^{odd}\right|}{\frac{1}{2}\left|I_{hkl}^{even}-I_{hkl}^{odd}\right|}.$

**Fig. 4:**
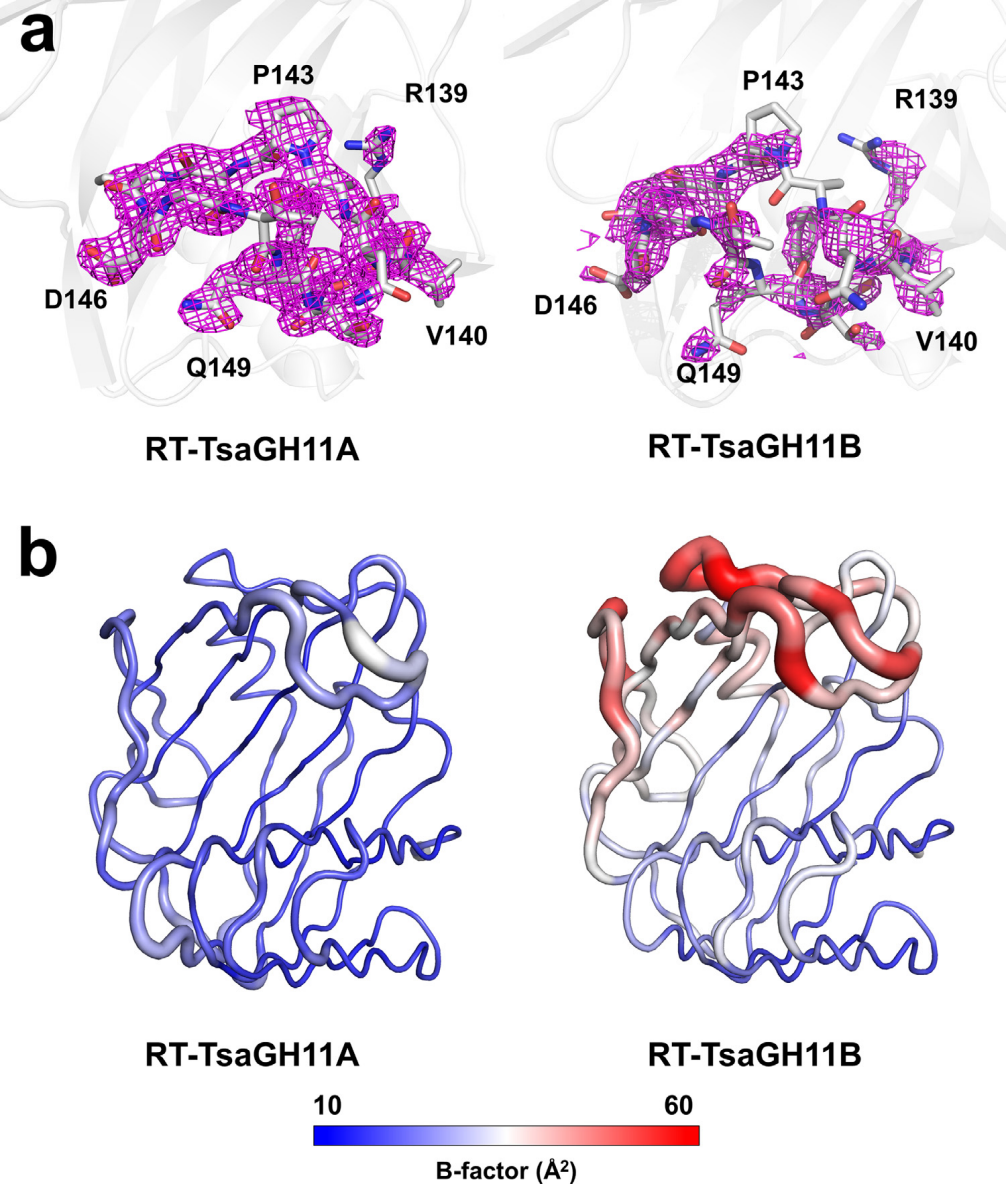
Room-temperature structure of TsaGH11 determined by SSX. (a) Fo-Fc omit electron density map (pink mesh, 3σ) of the determined thumb domain of TsaGH11. (b) B-factor putty representation of TsaGH11A and TsaGH11B as two molecules in an asymmetric unit.

The RT-TsaGH11 crystal contains two molecules in an asymmetric unit. In the crystal packing, the TsaGH11A molecule is surrounded by neighboring molecules, while the thumb and finger domains of TsaGH11B are exposed to the solvent. Due to their crystal packing, the two molecules exhibit different structural features. TsaGH11A displays an open conformation of the thumb and finger domains above the substrate-binding cleft with a B-factor value of 23.06 Å², whereas TsaGH11B displays an open conformation with a B-factor value of 36.42 Å² (Fig. 4). Notably, the electron density map showed that the thumb domain of SSX-TsaGH11B was largely disordered, differing from the crystal structure of TsaGH11B determined under cryogenic conditions. The structural information of TsaGH11 demonstrates the high flexibility of the thumb domain at room temperature and provides insights into the substrate recognition of xylanase GH11 (Fig. 4).

## 4 Experimental Design, Materials and Methods

The sample preparation, crystallization, data collection, and structure determination procedures of TsaGH11 have been described [1]. A codon-optimized TsaGH11 (UniProt: I3VTR8) gene for expression in *Escherichia coli* was cloned into the pBT7 vector (Bioneer, Daejeon, Korea) and transformed into *E. coli* strain BL21 (DE3). Protein purification was performed using a hexahistidine affinity column and size exclusion chromatography. Purified TsaGH11 protein was collected and concentrated to 20 mg mL^−1^ for crystallization. The crystallization screen of TsaGH11 was performed using the sitting drop vapor diffusion method at 22 °C using commercially available crystal screen kits. Microcrystals were obtained with a crystallization solution containing 0.1 M sodium acetate, pH 4.6, and 4.0 M ammonium acetate. Crystals suitable for the SSX experiment were further obtained using the hanging-drop vapor diffusion method at 22 °C. A crystal optimization experiment was performed on a 24-well VDX plate (Hampton Research, AlisoViejo, CA, USA) by mixing protein solution (2 µL) and reservoir solution (2 µL) and obtaining TsaGH11 crystals within 1 month.

Crystallization drops (total volume: ∼200 µL) were harvested using a pipette and transferred to a 1.5-mL microcentrifuge tube. This crystal suspension was left to stand at room temperature for 6 h, and then the supernatant (100 µL) was discarded. For the fixed-target (FT)-SSX experiment, sample delivery with a nylon mesh and enclosed film (NAM)-based sample holder was applied [9,10]. The crystal suspension (25 µL) was loaded onto the nylon mesh-based sample holder, which was further enclosed by a polyimide film (25 µm) to prevent sample dehydration.

X-ray diffraction data were collected at Beamline 11C at Pohang Light Source II (Pohang, Korea). The X-ray energy was 12,659 eV (wavelength: 9.7942 Å). The photon flux was ∼2 × 10^11^ photons/s. The vertical and horizontal X-ray beam sizes (full width at half maximum) focused by a K-B mirror were 3.5 and 8 µm, respectively. A NAM sample holder containing the TsaGH11 crystals was installed in the goniometer. The sample holder containing the TsaGH11 crystals was raster scanned at 50 µm intervals in the vertical and horizontal directions. Raster scanning was performed with 100 ms exposure with 0.022° oscillation. The diffraction data were collected at ambient pressure and 26 °C ± 0.5 °C. Diffraction data were recorded on a Pilatus3S 6 M detector with a 10 Hz readout. Images containing the Bragg peaks were filtered using Cheetah [7], with the following parameters: hitfinderAlgorithm = 8, hitfinderMinSNR = 5, hitfinderNPeaks = 20, hitfinderMinPixCount = 2, and hitfinderLocalBgRadius = 2. Diffraction patterns were indexed using CrystFEL (0.9.1 + 886ae521) [5] with the XGANDALF [11] indexing algorithm. During the indexing of Bragg peaks, the detector geometry was refined more than five times using a *geoptimiser* [12]. Indexed diffraction patterns were scaled with a *patialator* in CrystFEL [5]. The electron density map was obtained by molecular replacement method with MOLREP [13] program. Search model structure for MR was generated using Alphafold2 [14]. The model structure was constructed with the COOT [15] program. Structure refinement was performed using REFMAC5 [16] program. The geometry of the model structures was evaluated using MolProbity [17]. The protein structures were visualized and analyzed using PyMOL (DeLano Scientific LLC, San Carlos, CA, USA). The raw images have been deposited in Zenodo (http://zenodo.org). The structure factor and coordination have been deposited in the Protein Data Bank (http://rcsb.org).

## Limitations

Not applicable.

## Ethics Statement

This work meets the ethical requirements for publication in this journal. This work does not involve human subjects, animal experiments, or any data collected from social media.

## CRediT authorship contribution statement

**Ki Hyun Nam:** Data curation, Formal analysis, Validation, Visualization, Writing – original draft, Funding acquisition.

## Data Availability

Diffraction images (Original data) (Zenodo) Diffraction images (Original data) (Zenodo) Structure factor and coordinates (Original data) (Protein Data Bank)
